# Using machine learning to forecast conflict events for use in forced migration models

**DOI:** 10.1038/s41598-025-11812-2

**Published:** 2025-08-02

**Authors:** Yani Xue, Thomas Schincariol, Thomas Chadefaux, Derek Groen

**Affiliations:** 1 Department of Computer Science, Brunel University London, Uxbridge, UK; 2 Political Science, Trinity College Dublin, 2 College Green, Dublin 2, Ireland

**Keywords:** Agent-based modeling, Machine learning, Random forest, Migration, Simulation, Computational science, Environmental social sciences

## Abstract

Forecasting the movement of populations during conflict outbreaks remains a significant challenge in contemporary humanitarian efforts. Accurate predictions of displacement patterns are crucial for improving the delivery of aid to refugees and other forcibly displaced individuals. Over the past decade, generalized modeling approaches have demonstrated their ability to effectively predict such movements, provided that accurate estimations of conflict dynamics during the forecasting period are available. However, deriving precise conflict forecasts remains difficult, as many existing methods for conflict prediction are overly coarse in their spatial and temporal resolution, rendering them inadequate for integration with displacement models. In this paper, we propose a hybrid methodology to enhance the accuracy of conflict-driven population displacement forecasts by combining machine learning-based conflict prediction with agent-based modeling (ABM). Our approach uses a coupled model that combines a Random Forest classifier for conflict forecasting with the Flee ABM—a model of the movements of refugees and internally displaced persons (IDPs). The coupled model is validated using case studies from historical conflicts in Mali, Burundi, South Sudan, and the Central African Republic. Our results demonstrate comparable predictive accuracy over traditional methods without the need for manual conflict estimations in advance, thus reducing the effort and expertise needed for humanitarian professionals to provide urgent displacement forecasts.

## Introduction

Over the past decades, conflict, persecution, and violence have had profound effects on both the economy and society. The impacts of these factors include global food insecurity^1,2^, loss of life, infrastructure disruption, and forced displacement. By the end of 2024, the number of forcibly displaced people worldwide due to these factors reached 123.2 million (UNHCR: 2025^3^). Conflicts often last several years, leading to increased displacement and a growing need to support these displaced people in their countries of asylum, typically neighboring countries. Understanding and predicting the movement of people is crucial for policymakers. The allocation of resources—such as local support and material aid—by states or humanitarian organizations depends critically on the ability to accurately anticipate where and when displacement will occur.

Forecasting conflicts is essential in this regard, as the onset and escalation of conflicts are primary drivers of forced displacement. By accurately anticipating conflicts, policymakers and humanitarian organizations can better prepare for potential surges in displacement and ensure timely and effective allocation of resources such as food, shelter, and medical aid. This foresight enables the development of contingency plans and the establishment of support systems in regions likely to receive large numbers of displaced individuals. Additionally, early conflict forecasting can facilitate international cooperation and coordination, enhancing the overall capacity to manage migration flows and mitigate the humanitarian impact on affected populations.

Forecasting conflicts is inherently challenging due to their complex and multifaceted nature. Conflicts often arise from a mix of political, economic, social, and cultural factors, making accurate predictions difficult. The unpredictable nature of human behavior, involvement of multiple stakeholders with conflicting interests, and rapidly changing circumstances further complicate the process. Conflicts can escalate quickly and unexpectedly, driven by sudden events like political assassinations or economic collapses. Existing forecasting techniques, which rely on historical data and statistical models, often fail to capture the dynamic and non-linear nature of conflicts and struggle to incorporate real-time information and adapt to fast-paced developments. Traditional methods, such as regression models or time-series analyses, may not account for emergent behaviors and interactions between different actors in conflict scenarios.

Agent-based models (ABMs) offer a different approach by simulating the actions and interactions of individual agents to assess their effects on the system as a whole. This ability to model complex systems makes ABMs particularly suitable for migration modeling, as they aggregate the behavior of artificial agents and the interactions between them^4,5^. ABMs have been widely used in fields such as economics, biology, engineering, and social sciences^6^.

Yet, although various ABMs have been developed to model forced displacement^7,8^, few studies have incorporated conflict forecasting into their models. Recognizing this gap, our paper makes a novel contribution by coupling a machine learning-based conflict forecasting model with an ABM to predict forced migration. Specifically, we introduce a random forest (RF) classifier to forecast potential future conflict patterns, which then informs the Flee ABM^9^ to simulate primary displacement. In particular, the primary displacement could be either internal displacement or displacement of people to neighboring countries, while secondary displacement refers to the subsequent movements of those displaced people in neighboring countries.

We apply this hybrid approach to four real-world conflicts in Africa, taken from studies^10,11^, including the conflicts in Mali (2012), Burundi (2015), South Sudan (2013), and the Central African Republic (2013). Experimental results have shown its potential to improve the accuracy and relevance of migration predictions.

The key contributions of this paper can be summarized as follows:

1. A machine learning-based conflict forecasting model is proposed, where the RF model is trained on geographical information (e.g., terrain and altitude) and covariates (e.g., GDP per capita and population density) from the Peace Research Institute Oslo (PRIO), and conflict data from the Armed Conflict Location and Event Data Project (ACLED), using spatial and temporal inputs to predict conflict events. Our conflict forecasting model has demonstrated its capability of providing conflict forecasts on a daily basis, at the locality level, and spanning a couple of years.
2. A coupled model that reflects the relationship between conflict and forced population displacement is developed by integrating conflict forecasts into the Flee ABM. A simulation development approach is designed to construct, run and validate agent-based simulations of migration patterns, forecasting the number of displaced people over the simulation period.
3. The coupled model is validated using case studies from historical conflicts in Mali (2012), Burundi (2015), South Sudan (2013), and the Central African Republic (2013). Experimental results demonstrate that the coupled model achieves better predictive accuracy than the original Flee ABM, used in^10,11^. Furthermore, our model shows potential for application in real-world forecasting contexts, where ground truth conflict progression information does not exist and simulations cannot be done in the conventional way.

## Related work

### Conflict forecasting

The prediction of future conflicts is of both substantive and practical importance for governments and non-governmental organizations in humanitarian operations. Recently, research on conflict forecasting has drawn increasing attention. Expert knowledge, often provided by political analysts and pundits, offers valuable insights into future conflict based on their research and personal experience^12,13^. However, expert predictions on geopolitical events have been shown to lack accuracy and, in some cases, perform no better than random guesses^12^.

The recent use of machine learning techniques in conflict forecasting marks a considerable evolution beyond traditional statistical models. Classic methods such as logistic regression have the advantage of interpretability, but often fall short in capturing the multidimensional and non-linear relationships intrinsic to conflict^14–16^. Machine learning algorithms such as RF, Support Vector Machines, and Neural Networks have been increasingly adopted for their superior predictive accuracy, especially in the context of voluminous and imbalanced data^12,17,18^. These models have been employed to predict various outputs, including the onset of conflict, changes in conflict intensity, fatalities, and even specific types of conflict such as genocide or civil unrest^19–21^.

Various conflict-related events have been of interest. Some studies focus on predicting the onset of conflict within specific geographic units and time frames, while others aim to forecast changes in conflict intensity, measured through variables such as the number of fatalities or incidents^22–24^. More targeted research has looked at predicting specific types of events like genocide, using features that encompass not just economic or political instability but also indicators like hate speech or ethnic tensions^20,25^.

High-quality data are of vital importance for these conflict forecasting approaches in producing timely and truly useful forecasts. A number of widely used conflict datasets, such as the Uppsala Conflict Data Program (UCDP, https://ucdp.uu.se/downloads/)^26^ and the Armed Conflict Location and Event Dataset (ACLED, https://www.acleddata.com/data^27^ provide very detailed and regularly updated information on conflict events. These datasets have been extensively applied in conflict forecasting studies, such as ViEWS (Violence Early Warning System), which provides conflict predictions at the country and subnational level^23^ and ABSCIM (Agent-Based Simulator for Conflict-Induced Migration), which integrates a conflict model forecasting discrete daily conflict events^28^.

Combining ABMs with machine learning (ML) methods is becoming increasingly common^29^. ML techniques can be integrated into ABMs for various purposes. For example, they can assist in data processing and model calibration. Modeling accuracy can be enhanced through reinforcement learning, or data preprocessing can be performed using decision trees^30^. Moreover, ML can help estimate micro-level variables within ABMs, rather than focusing only on macro-level system behavior, as demonstrated in^31^ in the context of the housing market. Conversely, deep learning models can be trained on data generated by ABMs, as shown in^32^, which explores the interplay between conflict and disease spread. ML models can also provide real-time predictions in ABM frameworks^33^, enabling established models, such as the Flee model, to run in real time without requiring observed data. Although ML-driven conflict forecasting has not yet been fully integrated into ABMs, related spatial methods have been applied in conflict studies, including join count statistics^34^ and the Langevin diffusion equation^35^. Additionally, more traditional machine learning techniques have been employed to address discrepancies in data frequency, for example, when aligning conflict and climate data to infer causal relationships^36^. ML methods have also been used to predict fine-grained patterns of violence, as demonstrated in the cases of Colombia and Indonesia^37^. These studies explore fine-grained geographic scales but remain coarse in the temporal dimension, typically operating at the yearly or monthly level.

### Forced migration modeling

Forced migration is a complex social phenomenon, often driven by conflicts in the country of origin^38,39^. Agent-based modeling is a popular simulation approach for forecasting the movements of forcibly displaced populations. In existing ABMs for conflict-driven forced migration, modeling approaches for future conflict events are often simplistic, relying mainly on expert knowledge or some basic approaches. For example, Suleimenova et al.^40^ developed an ABM for the Tigray Crisis, where the generation of future conflict progression was according to the description of domain experts from Save The Children^41^. In addition, Groen et al.^42^ developed a hybrid model that combines a conflict generator, Flare, with an existing forced migration model. The Flare uses a highly stochastic algorithm to randomly generate conflict events and predict conflict events over time, while the migration model integrates conflict information as input to model refugee movements. Since the evolution of conflict events is artificially generated, it limits the models’ ability to accurately reflect the dynamics of real-world conflicts and their impact on migration patterns.

Recent advances have developed various approaches for integrating conflict forecasting into ABMs. For example, Mehrab et al.^28^ proposed an agent-based framework to predict conflict-induced migration, which utilizes a Log-Cox Gaussian point process model with a spatial mesh to generate future conflict events. One limitation of this framework is that it can predict future daily refugee flows but not their destination. Searle and van Vuuren^43^ proposed a generic framework for aiding in the development of ABMs to forecast the movement of refugees, undocumented migrants and IDPs, where the conflict progression is modeled based on the rules of a typical cellular automata. However, the simulation lacks systematic validation and is limited to predicting refugee arrivals in a neighboring country at the country level.

Although a number of ABMs have been designed for forced migration^28^, it is crucial to enable rapid development to support timely humanitarian responses to conflict-induced displacement, given a particular conflict situation. The general simulation development approach (SDA), presented in^10^, provides a structured methodology for developing simulations in conflict scenarios, ensuring rapid simulation development. SDA comprises six phases: selecting a specific conflict scenario, collecting relevant data from various repositories such as UNHCR, ACLED and Bing Maps, constructing a network-based ABM, refining the model with additional inputs such as population data and border policies, executing simulations using the Flee simulation code, and validating results against the full UNHCR refugee numbers. This approach was tested in three different historical conflict scenarios, achieving over 75% accuracy in predicting destinations in these cases. Its scalability in different conflict scenarios and flexibility in integrating diverse data sources make it particularly suitable for future conflict scenarios. Nevertheless, this approach cannot predict population displacement in future conflicts, highlighting the need to integrate advanced machine learning-based conflict forecasting tools into well-established ABMs, such as the Flee model.

## Methods

### Flee ABM

This study employs the Flee^9^ model to predict conflict-driven population displacement due to its ability to rapidly develop simulations for conflict scenarios following the SDA process in^10^. The Flee model has been applied in real-world scenarios, such as the Tigray crisis (2020)^40^, to support Save The Children by simulating the distribution of displaced people. As aid budgets are usually limited, by understanding refugee movement patterns, governments, organizations and non-governmental organizations can save time in the response phase and improve the targeting of aid^41^.

**Fig. 1:**
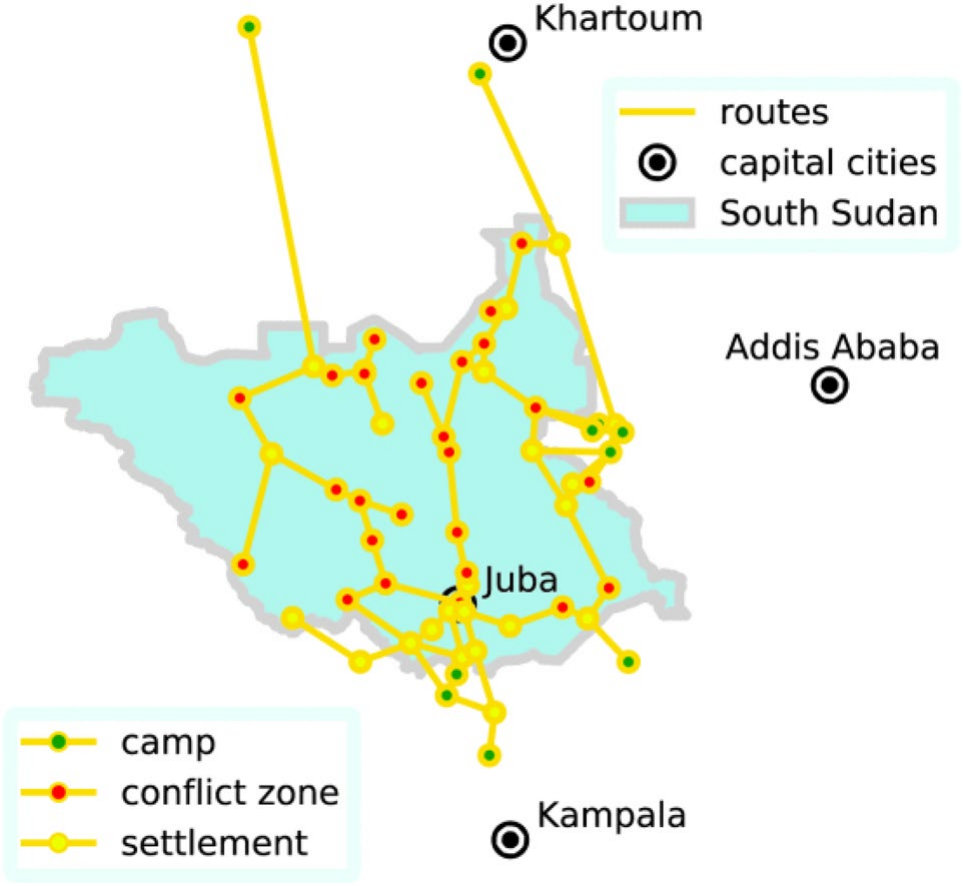
Graphical overview of South Sudan conflict location graph (58 locations and 69 routes)^45^.

In the Flee model, people fleeing a conflict are modeled as agents, with their decision-making processes simulated using a predefined ruleset. These agents interact within a location graph that represents the spatial environment. Figure 1 illustrates a location graph for a historical South Sudan conflict, comprising 58 locations and 69 routes, where each vertex denotes a location, such as conflict zones, camps, or major settlements. During the simulation period, the Flee code spawns a certain number of agents in conflict zones, who then navigate the location graph on a daily basis to search for camps/settlements. As a baseline, we have adopted the assumption in the latest version of the Flee code (https://github.com/djgroen/flee/releases/tag/v3.1)^44^. Supplementary Table 1 presents the simulation configuration settings and assumptions for this study. Please refer to^10^ for more details. It is worth mentioning that the Flee model utilizes conflict progression data based on ground truth (ACLED conflict data). ACLED conflict data are converted into input for the simulation runs, using binary numbers to record whether a conflict event occurred at a specific location each day. This conversion is based on the assumption that once a conflict starts at a specific location, it will continue until the end of the simulation (Fig. 1).

### Conflict forecasting model

We adopt two different methods to predict conflicts. One method aims to forecast the real conflict events at the daily level in a binary classification of conflict/no conflict. The other method focuses on identifying the initial onset of conflict in each locality and considers it as a continuous conflict zone until the end of the simulation. The Flee model was originally designed with this method. Finally, we compare both forecasting methods to determine which one aligns better with the needs of the Flee model. Both models use the same input data.

*Data*. Our conflict forecasting model includes three types of inputs. The first component is derived from the PRIO-GRID v.2.0 dataset (available at https://grid.prio.org/#/download), which provides geographical information supplied by the Peace Research Institute Oslo (PRIO). The PRIO-GRID data is a geo-referenced dataset that standardises a wide range of variables potentially influential to conflict at a granular geographic level. These variables may include indicators related to demography, political stability, and economic conditions, among others. The PRIO-GRID data incorporates both static and dynamic variables. Static variables include factors that are relatively constant over time, such as terrain and altitude, administrative boundaries, and the distribution of ethnolinguistic groups. Dynamic, or yearly, variables capture changes in indicators like GDP per capita, governance quality, population density, as well as conflict history and social variables. For each target location, we assign the covariate values based on the PRIO-GRID cell in which the target is located.

The second component of the input relates to spatial diffusion. In particular, the model collates the occurrences of conflicts in the other locations of the study for two distinct periods: the past week and the past month. This aggregation serves two key functions. First, it captures short-term fluctuations in conflict patterns, enabling the model to adapt to sudden escalations or de-escalations at a larger geographical scale. Second, it incorporates the longer-term context of a month to uncover any trends or cycles.

Finally, the third input is an autoregressive element that incorporates data from the last 31 days to indicate the presence or absence of a conflict event in the location of interest. For each day within the preceding 31-day period, a binary value is assigned to represent whether or not a conflict event occurred in that specific geographical location. The conflict information is extracted from the ACLED event dataset, available through the Data Export Tool (https://acleddata.com/data-export-tool/), by selecting events within a 0.1$^{\circ }$ buffer around each locality’s longitude and latitude (approximately 11 km at the equator). This array of binary values forms a temporal sequence for each conflict zone, effectively capturing the recent history of conflict events or non-events in that area. This enables the model to identify recurring patterns or seasonality in conflict events.

The outcome of the conflict forecasting model is a daily prediction for each location to allow the network-based migration model to run properly.

An issue often encountered in conflict data is the class imbalance between ‘events’ and ‘non-events’. To address this, a specific proportion of ‘non-event’ observations is randomly sampled. These sampled ‘non-events’ are then amalgamated with ‘event’ observations to form a balanced subset of features. This procedure ensures that the learning algorithm is not biased toward the majority class, thereby enhancing its predictive accuracy.

*Algorithm*. The algorithm used for the predictive modeling task is the RF classifier. This algorithm is particularly well-suited for the problem at hand due to its capacity to model complex, non-linear relationships while avoiding overfitting. The decision to use only tree-based models, such as RF, is driven by the specific challenges of the conflict forecasting task. Tree-based models are well-suited for handling high-dimensional data and imbalanced classes, whereas other machine learning models, such as Support Vector Machines, have limited performance in such settings. This has been a topic of ongoing discussion in the conflict forecasting literature^17,46^. Moreover, leading models in the field have adopted RF frameworks^23^.

**Fig. 2:**
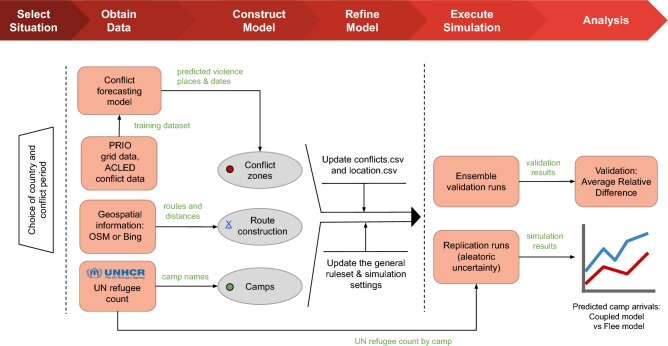
Simulation development framework of the coupled model for predicting refugee migration patterns.

The foundation of the RF model is justified by the exacting specifications of the Flee model’s input prerequisites. The necessary forecasts are expected to be on a daily basis, at the locality level, and spanning a couple of years. The substantial temporal and spatial granularity poses a significant challenge in designing the model, making it exceptionally complex. In contrast, existing models in the literature typically operate at a monthly-country level, with a maximum horizon generally up to 12 months (Fig. 2).

The RF classifier is trained on the balanced subset of features, which includes not only the transformed autoregressive sequences but also the spatial information and covariates for each grid cell. The labels for training, in this case, indicate the presence or absence of a conflict event. Specifically, the model was trained on conflicts in Mali (2012), the Central African Republic (2013), Burundi (2015), and South Sudan (2013). The code for the algorithm is publicly available at: https://github.com/ThomasSchinca/conflict_forecast_colab.

### Coupled model

We propose a framework to aid in the design of a coupled model that integrates conflict forecasts generated by the RF classifier into the Flee ABM for simulating conflict-driven population displacement, as illustrated in Fig. 2. This framework, which is based on the generalized SDA introduced by Suleimenova et al. in^10^, provides a structured approach for constructing, refining, executing and validating forced displacement simulations. Our framework consists of six phases: (i) *Select Situation*, (ii) *Obtain Data*, (iii) *Construct Model*, (iv) *Refine Model*, (v) *Execute Simulation*, and (vi) *Analysis*.

The *Select Situation* phase involves defining the geographic and temporal context of the simulation. We select a country where conflict has led to large-scale forced migration and an appropriate time period. The time period of the simulation could be: (i) a historical time period, to support retrospective validation using observed refugee data; (ii) a future time period, to predict the number of refugees in camps over time; or (iii) a combination of both, to simulate an ongoing conflict across past and future periods for both validation and prediction purposes.

The *Obtain Data* phase focuses on acquiring relevant data for both the conflict forecasting model and the Flee model:

- For the conflict forecasting model, relevant historical conflict event details are derived from ACLED, while geographical information and covariates are obtained from PRIO. Then, the RF classifier is trained on historical data over the last five years before the start of a conflict period. This model “predicts” conflict events in the historical conflict period, and its performance is evaluated based on the real conflict progression dataset. This model is fine-tuned to find the best balance between precision and recall, by optimising its performance on both accuracy and the ability to capture conflict events.
- For the Flee model, we obtain predicted conflict progression generated by the conflict forecasting model, along with information on camp names and refugee counts sourced from the United Nations High Commissioner for Refugees (UNHCR) database (https://data2.unhcr.org/en/situations), and geospatial information extracted from OpenStreetMap (OSM) platform (https://www.openstreetmap.org) or Bing Maps plaform (http://www.bing.com/maps) for constructing the location graph.

In the *Construct Model* phase, an initial coupled model is constructed by integrating conflict forecasts generated by the conflict forecasting model into the Flee model. The initial coupled model is defined as a network-based ABM in comma-separated values (CSV) format, representing conflict zones, camps, and towns, along with routes connecting them. Conflict forecasts are stored in a conflicts.csv file, which records daily conflict events at various locations during the forecast period and serves as input for the Flee model. This file I/O approach enables seamless integration of conflict forecasts into the simulation. For further details on the model construction process and CSV formats, please refer to^47^.

In the *Refine Model* phase, the coupled model is then refined to satisfy different conflict scenarios. This involves configuring the conflicts and locations for running simulations and updating the general ruleset and simulation settings.

The *Execute Simulation* phase involves a series of simulation runs to produce main simulation results: (1) ensemble validation runs, which utilize an automated ensemble execution function in FabFlee, a plugin of the FabSim3 automation toolkit^48^, to compare the outputs of these replicas against observed UNHCR validation data and automatically generate validation results^44^; and (2) replication runs for aleatoric uncertainty, which perform ensemble runs with multiple replicated instances (replicas) to produce predictions to account for aleatoric uncertainty in the coupled model.

Lastly, in the *Analysis* phase, we use these results to extract the accuracy of forced migration forecasts and aleatoric uncertainty. In addition, to investigate whether the coupled model can achieve comparable accuracy to the original Flee model, we compare the predicted daily camp arrivals generated by the coupled model with those produced by the original Flee model.

## Results

We conducted case studies on four historical African conflicts: (1) The Mali conflict with a simulation period of 300 days from 29th February 2012, (2) Burundi conflict with a simulation period of 396 days from 1st May 2015, (3) South Sudan conflict with a simulation period of 604 days from 15th December 2013, (4) Central African Republic conflict with a simulation period of 820 days from 1st December 2013. While these conflicts started in different contexts, they share common driven factors, such as violence political instability and civil war, which have led to large-scale displacement. The chosen time periods capture the critical phases of each conflict, where major forced displacement crises occurred, and ensure the availability of reliable data for model validation. The conflict instance for each country is denoted as Mali 2012, Burundi 2015, South Sudan 2013, and CAR 2013, respectively.

### Daily conflict/no conflict prediction using the RF model

Our initial analysis reveals a significant imbalance in our dataset, with over 95% of the data points representing periods of peace (value 0) across all four scenarios. This imbalance poses a challenge for our forecasting task, particularly because we need to predict conflict for various time horizons, ranging from one year to several years into the future. It is particularly problematic because our training data is heavily skewed towards peace (value 0), while the test data is less skewed. This discrepancy leads to the model underpredicting conflicts (value 1) across different time horizons.

To address this challenge, we employ a downsampling technique on the training dataset, where we randomly omit a portion of the peaceful events. The downsampling factor was determined through an optimisation process, aiming to strike a balance between accuracy and recall. Accuracy tends to be exceptionally high if we exclusively predict peaceful events, considering their preponderance in the dataset. Conversely, recall provides insight into how effectively we predicted conflict-related events. Our objective is to strike the optimal balance aligned with the requirements of the migration model. Supplementary Fig. 1 displays the metric’s performance under various downsampling values. In this graph, the downsampling factor ‘*k*’ corresponds to the reduction factor applied to the training dataset featuring zero values. Our analysis indicates that the most favourable compromise is achieved by downsizing the peaceful observations by a factor of 20, corresponding to $k=20$.

We evaluate the performance of the RF Classifier by comparing it against two baseline models, both based on a Bernoulli distribution. The first model uses a Bernoulli distribution with $p=0.5$, simulating a coin toss. The second model uses a Bernoulli distribution with *p* equal to the proportion of 1s in the actual data, reflecting the true distribution of observed values.

**Fig. 3:**
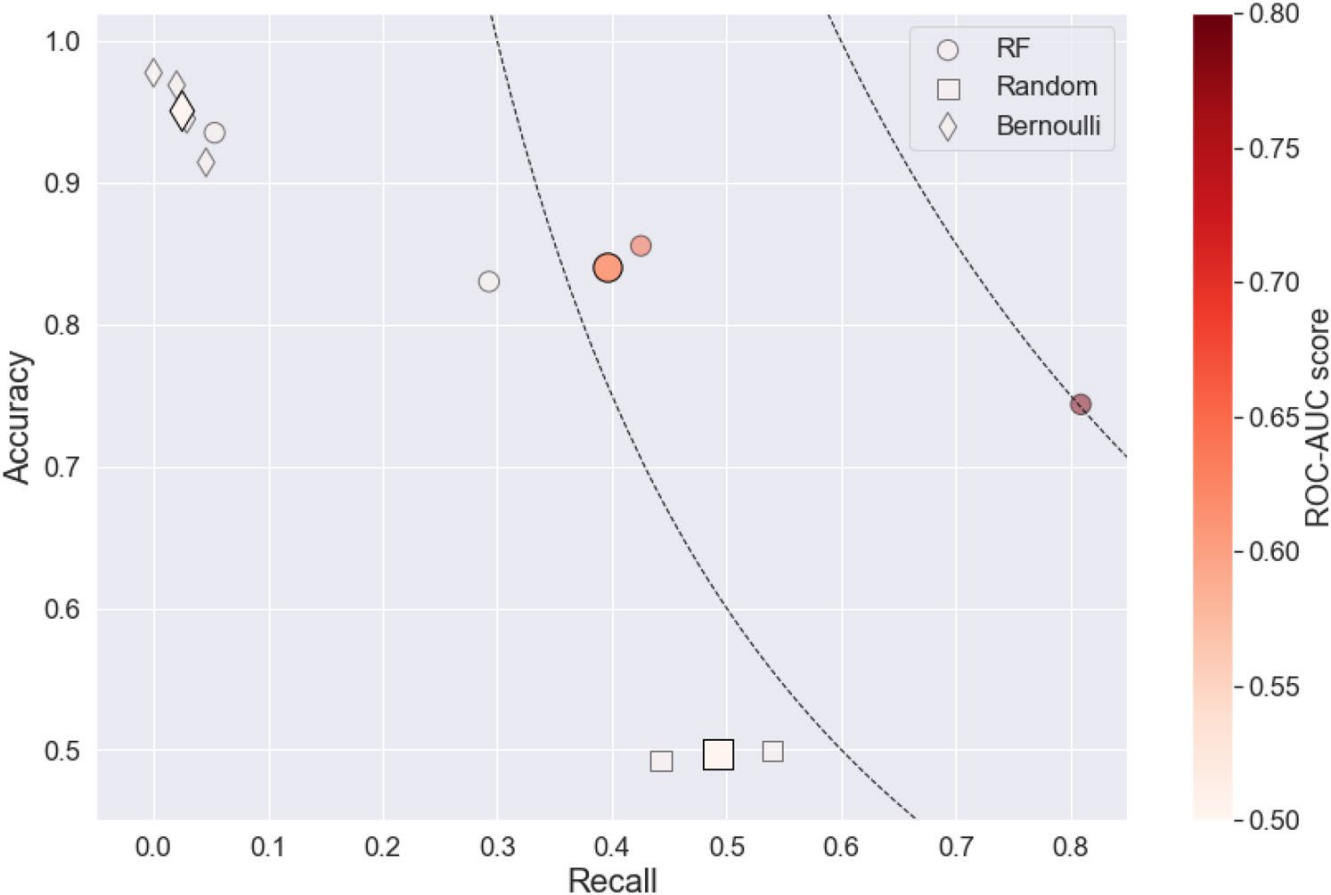
Comparison of recall vs accuracy across models: the circle shape represents the RF model, the diamond shape a Bernoulli distribution, and the square shape a random guess. The plain shape represents the mean value and the transparent markers represent the four simulations for each model. The ROC-AUC score is illustrated by a gradient red color, where a deeper red shade indicates a higher score. The dotted line is the product of accuracy and recall when equal to 0.3 and 0.6.

Figure 3 shows the recall, accuracy, and ROC-AUC score across three conflict forecasting models (RF model, Bernoulli distribution, and random guess). The ROC curve score measures how well a model can tell the difference between conflict and no-conflict cases. It measures the area under the curve of the true positive rate against the false positive rate. A score of 1 means perfect predictions. A score of 0.5 or lower means the model is no better than random guessing (Fig. 3).

As can be seen, the RF model consistently outperforms other models, achieving the highest scores in both the product of recall and accuracy, as well as in the ROC-AUC metric (see Supplementary Table 2 for more detailed results). In contrast, the Random and Bernoulli models exhibit low standard deviations in accuracy and recall across all study cases, with their observations clustered around specific points: (0.5, 0.5) for the random guess and (0, 1) for the Bernoulli model. These patterns can be attributed to the inherent characteristics of each model. The Bernoulli model, predicting mostly zeros, achieves high accuracy in unbalanced datasets but struggles to correctly identify conflict events (low recall). The random guess, by definition, predicts an equal proportion of ‘1’s and ‘0’s, resulting in scores of approximately 0.5 for both recall and accuracy. The RF model, however, demonstrates more varied performance across cases. It achieves excellent scores for Burundi (with a product of recall and accuracy around 0.6), average performance for Mali and the Central African Republic (CAR), and a low recall score for South Sudan—though still twice as high as the Bernoulli model (0.053 compared to 0.026). The RF model’s ROC-AUC scores further highlight its superiority, particularly in the case of Burundi where it achieves a score of 0.78. For CAR, the RF model shows a slight improvement (0.65), while for Mali and South Sudan, its performance is comparable to that of the Random and Bernoulli models. This analysis underscores the RF model’s potential for conflict prediction, especially in certain contexts, while also highlighting the variability in its performance across different cases.

**Fig. 4:**
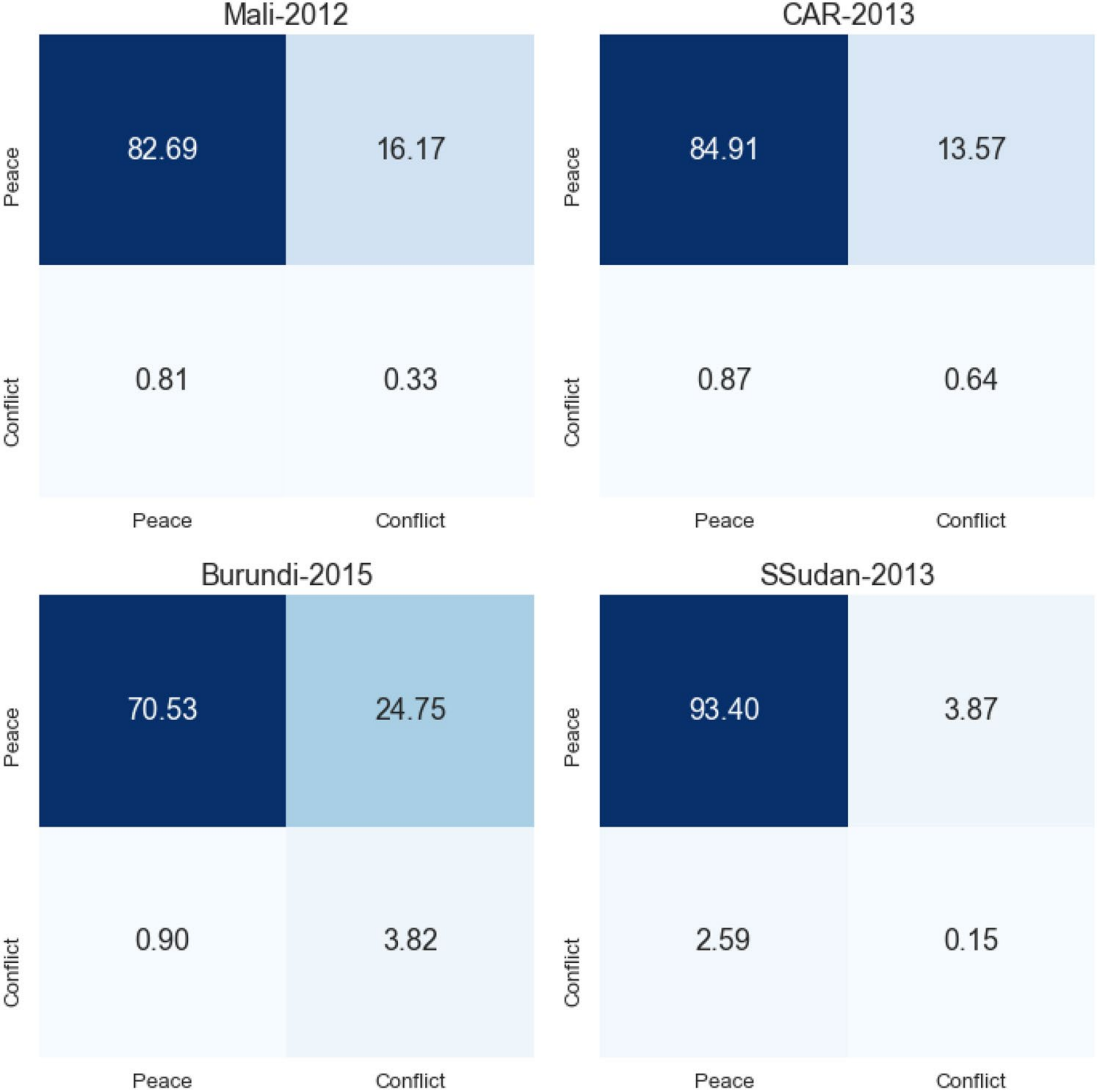
Confusion matrices in percentage for the four studied cases. Top-left: Mali 2012, top-right: Central African Republic 2013, bottom-left: Burundi 2015 and bottom-right: South Sudan 2013; predicted in x-axis and actuals in y-axis.

The forecasting classification confusion matrices are displayed in Fig. 4. In the four cases, we are overpredicting conflict events, or we have high values of false positives. However, the majority of cases are predicted as peaceful, especially in South Sudan in 2013, with 93% of True negative cases. Only Burundi has slightly higher false positive rate with 24% of the cases, compared to 16% and 13% for Mali and Central African Republic. This can be explained by the slight difference in the peaceful event distribution, as Burundi has a slighter lower percentage around 95% when the mean value is 97%. It is also the studied case with the higher True positive rate with almost 4% of the observations. On the other side, the RF model has high false negative value with South Sudan 2013 with around 2.5% and only 0.15 % of true positive observations.

The favourable outcomes observed in Burundi may be attributed to the relatively short prediction horizon (approximately 1 year) and the limited number of locations (7). Conversely, South Sudan presents a more challenging scenario with 25 localities to forecast and an extended prediction period of almost 2 years. Additionally, in the 5 years preceding the prediction period, 5 localities (constituting 20% of the total) accounted for approximately 50% of the conflict events. This concentration could lead our model to centralize predictions around these areas and potentially overlook the broader distribution of conflict events. Similar patterns emerge in the Central African Republic, where our model accurately forecasts high-conflict localities but may miss the dispersion in others. Lastly, in Mali, the scarcity of conflict events (approximately 1% of the overall test set) prompts the RF model to overpredict.

We also evaluated eXtreme Gradient Boosting (XGBoost) as an alternative to the RF model for daily conflict/no-conflict prediction over a future time period. As shown in Supplementary Table 3, XGBoost performs worse than the RF model in terms of recall, accuracy, and the ROC-AUC metrics. Additional analysis on the impact of the forecast horizon on the classification metrics is presented in the Supplementary Fig. 2. As expected, the score decreases with time in the Burundi and CAR examples. In Mali and South Sudan, time brings more and more fluctuations in the F1-score, showing increasing uncertainty in the predictions.

### First conflict onset prediction using the RF model

We use the RF model to predict the time until the first conflict onset at the locality level, measured in days over a future time period. Once a conflict is predicted to begin in a specific region, we assume that it will persist for the remainder of the simulation period. This assumption simplifies the model while still capturing the often protracted nature of local conflicts.

Similarly to the daily conflict/no conflict prediction, we encounter a challenge with non-events, which in regression results in a higher value for the upcoming onset in terms of the number of days. To tackle this issue, we also implement a downsampling technique for the outputs that extend beyond 120 days in the training set. To evaluate the performance of the RF model, we use the same benchmark models, Bernoulli distribution and random guess, as in the daily conflict/no conflict prediction. We simulate when each model makes its first ‘conflict’ prediction. The day this happens is recorded as the predicted onset of conflict.

**Fig. 5:**
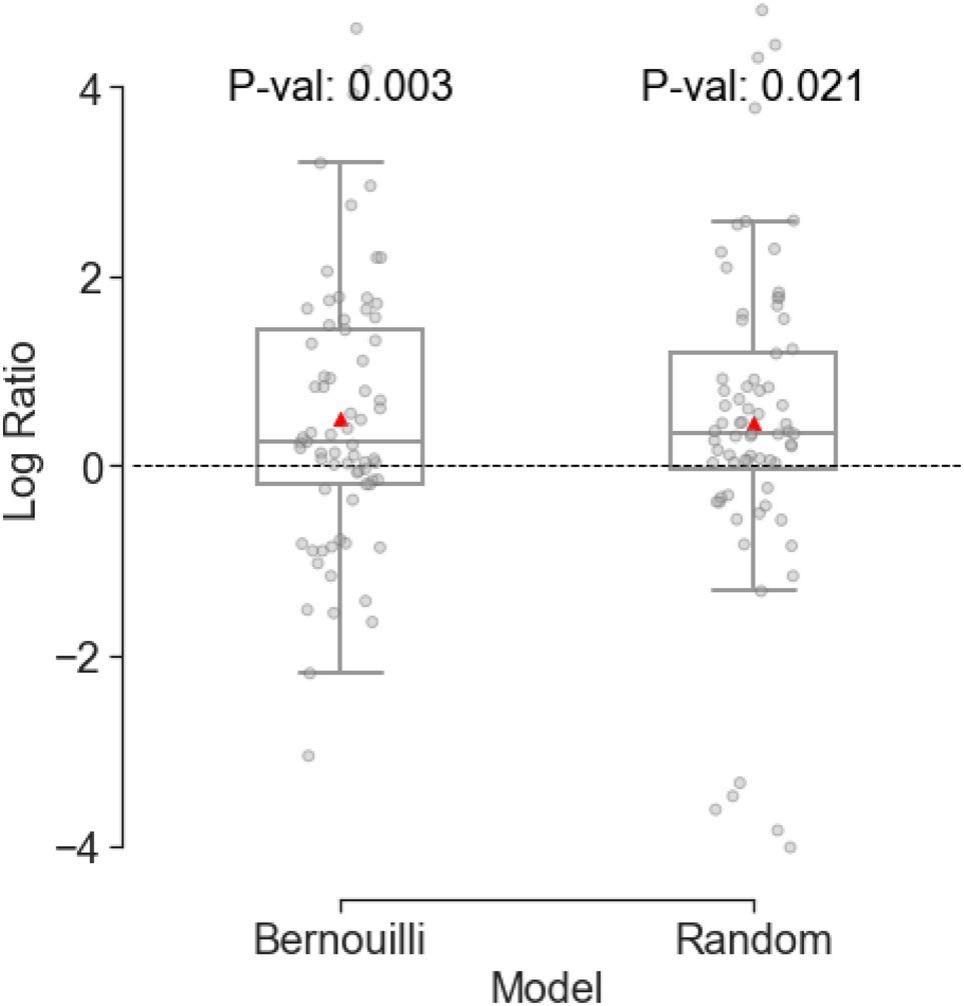
Boxplot comparing the log ratios of two models, Bernoulli and random guess, with the RF model. Each point represents an individual data sample. The red triangles denote the mean value of MSE log ratio for each model. The p-values indicate the statistical significance of the difference between the log ratios for each model from 0. A positive log ratio implies a higher MSE, and worse performance for the benchmark model compared to our model.

To evaluate the forecast results, the log ratio of the Mean Squared Error (MSE) of the benchmark model over the RF model is plotted in Fig. 5. As can be seen, our model has significantly better results than the two benchmark models. A positive log ratio implies a higher MSE, and hence worse performance for the benchmark model compared to our model. We find that 66% of the localities (represented by grey points) have a positive log ratio for the Bernoulli model (with *p* the proportion of one in the data), while 75% for the Random model. The mean values of the log ratios are 0.51 and 0.47, respectively. The statistical evaluation highlights this superiority, with a *p*-value of 0.003 for the Bernoulli model and 0.02 for the random model, derived from a t-test on the log ratio, testing its deviation from zero.

The XGBoost model was also tested for the first conflict onset prediction. Supplementary Fig. 3 plots the log ratio of MSE of the benchmark models over XGBoost. As can be seen, the model’s log ratios with benchmark models were not significantly different from zero. One potential explanation is the tendency of XGBoost to overfit, especially with the noisy fatalities covariates. Unlike XGBoost, RF has unrelated random trees, which avoid the risk of overfitting.

### Conflict-driven population displacement simulation

To evaluate the added value of two types of conflict progressions generated by the RF model in predicting the displacement of people, we compared our method with the Flee model presented in^10^. We constructed three simulation instances, each corresponding to a different conflict progression input: (1) Flee (recorded conflict), where conflict progression is based on the ground truth (i.e., ACLED conflict data) as used in^10^; (2) Flee (predicted conflict [RF-daily]), where conflict progression is generated by daily conflict/no-conflict prediction using the RF model; and (3) Flee (predicted conflict [RF-onset]), where conflict progression is generated by first conflict onset prediction using the RF model.

The accuracy of three simulation instances was assessed by the Average Relative Difference (ARD) metric, which is calculated as follows:

1 $$\begin{aligned} E(t) = \frac{\sum _{x\in S} (|n_{sim,x,t} - n_{data,x,t}|)}{N_{data,all,t}} \end{aligned}$$

where $n_{sim,x,t}$ denotes the number of refugees predicted by a simulation in each camp *x* of the set of all camps *S* at time *t*, $n_{data,x,t}$ denotes the observational data from UNHCR for each camp *x* of the set of all camps *S* at time *t*, and $N_{data,all,t}$ is an aggregation of the observational data from UNHCR for all camps at time *t*. The ARD is a linear error measure which complies that every mismatch in the estimation of a human arrival should contribute equally to the error score. As a result, both an overprediction and an underprediction of arrivals by $100\%$ would result in an ARD score of 1.0. An ARD value of 0.0 indicates that a simulation is completely in line with the validation data (0% error). It is possible for ARD values to be higher than 1.0. This occurs when the Flee model overpredicts actual arrivals by more than $100\%$, a phenomenon that frequently occurs in very early stages of an armed conflict. For ease of communication and consistency with previous literature, we will indicate a simulation to have an error of $50\%$ when the ARD is 1.0 and an error of $0\%$ when the ARD is 0.0.

To configure simulations using the Flee code, we modified the default assumptions, resulting in two different rulesets: ruleset 1.0, which follows the assumptions proposed in^47^, and ruleset 2.0, which provides a more realistic version by incorporating additional movement rules to simulate more complex behaviors. See supplementary materials for a description of the two rulesets.

Similar to many other simulation codes, Flee is non-deterministic, resulting in variations in results with each execution. To reduce the impact caused by aleatoric uncertainty, we execute 100 replicas of each individual simulation. To reduce the execution time for all conflict instances, we apply the FabFlee automation tool and a pilot job mechanism, i.e., QCG-PilotJOB^49^, to efficiently run ensemble forecasts on the ARCHER2 supercomputer.

Table 1 presents the ARD results of all simulation instances in four conflict scenarios. To interpret these results, we need to acknowledge that a conflict forecast that exactly corresponds to the recorded ACLED data would result in a near-perfect match in ARD scores, as only aleatoric uncertainty of the probabilistic Flee algorithm, which is typically around 0.5%, would introduce noise in the results. Therefore, when this ARD difference is systematically very small, we can choose to produce forecasts reliably using the Flee model without having to rely on ACLED data (which covers only historical events).

**Table 1:** ARD values of three simulation instances for four conflict scenarios.

**Ruleset**	Conflict name	ARD	Flee (recorded conflict)	Flee (predicted conflict [RF-daily])	Flee (predicted conflict [RF-onset])
Ruleset 1.0	Mali 2012(300 days)	Mean	0.3576	0.2021	0.2133
		Std	1.60E-03	8.53E-04	3.15E-03
	Burundi 2015(396 days)	Mean	0.5595	0.4837	0.4535
		Std	1.23E-03	3.24E-03	1.79E-03
	South Sudan 2013(604 days)	Mean	0.4603	0.3147	0.3819
		Std	1.99E-03	1.33E-03	2.97E-03
	CAR 2013(820 days)	Mean	0.2930	0.4497	0.3586
		Std	6.95E-04	8.89E-04	4.99E-04
Ruleset 2.0	Mali 2012(300 days)	Mean	0.3577	0.1827	0.1951
		Std	1.81E-03	1.41E-03	2.89E-03
	Burundi 2015(396 days)	Mean	0.4402	0.3861	0.3131
		Std	1.52E-03	2.52E-03	1.98E-03
	South Sudan 2013(604 days)	Mean	0.4329	0.3361	0.3767
		Std	1.85E-03	9.25E-04	3.06E-03
	CAR 2013(820 days)	Mean	0.2743	0.4168	0.3441
		Std	4.65E-04	7.19E-04	4.45E-04

As shown in Table 1, the ARD values range between 0.25 and 0.6 in all cases, which means that the simulation is at least 70% correct relative to the UNHCR data in all. Among the three simulation instances, both Flee (predicted conflict [RF-daily]) and Flee (predicted conflict [RF-onset]) obtain lower ARD values (i.e., better validation scores) than Flee (recorded conflict) in all conflict scenarios except for CAR 2013. Flee (predicted conflict [RF-daily]) outperforms Flee (predicted conflict [RF-onset]) in Mali 2012 and South Sudan 2013, while it performs worse in the remaining cases. The change from Ruleset 1.0 to the more realistic Ruleset 2.0 results in lower ARD values in most scenarios, which indicates that more realistic movement assumptions have a positive impact on the simulation of conflict-driven population displacement. Moreover, the three simulation instances show relatively low standard deviations of ARD, indicating the robustness of the predictions. In summary, simulations incorporating predicted conflict progressions achieve comparable accuracy to those using recorded conflicts. Namely, the coupled model is effective in modeling conflict dynamics even without using ACLED data, providing an alternative to forecast future conflicts.

**Fig. 6:**
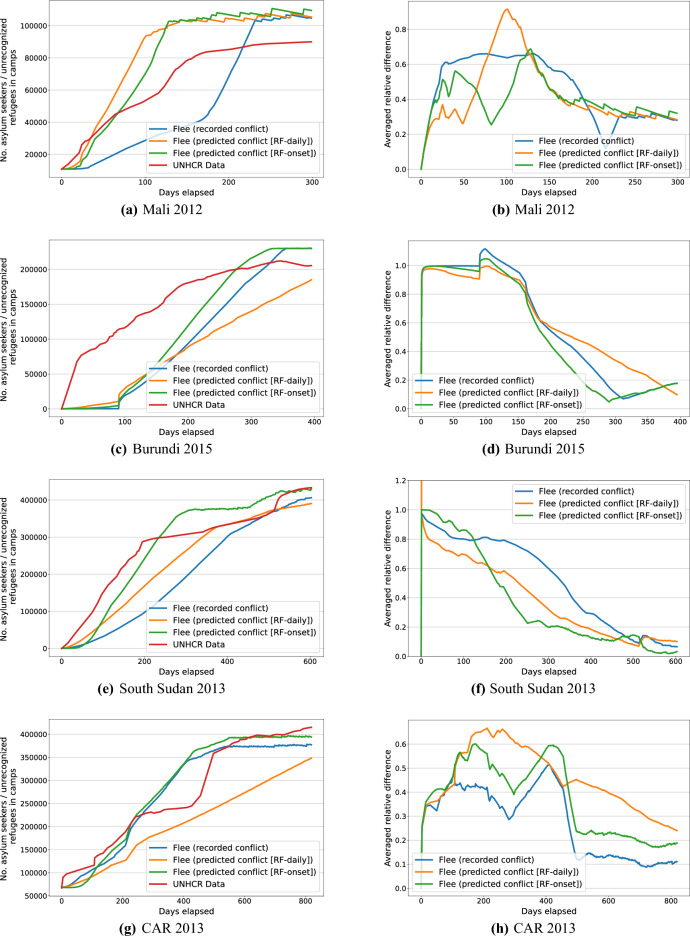
The number of camp populations predicted by three simulation instances in a single simulation run and observed data from UNHCR (left), and ARD values for simulation results compared to observed data from UNHCR (right) under ruleset 1.0 for the four conflict scenarios. Blue line: Flee (recorded conflict), yellow line: Flee (predicted conflict [RF-daily]), green line: Flee (predicted conflict [RF-onset]), red line: observational data from UNHCR.

In Fig. 6, we present the daily number of arrivals in camps predicted by three simulation instances in a single simulation run and observed data from UNHCR (Fig. 6a, c, e, g), and ARD values for simulation results compared to observational data from UNHCR (Fig. 6b, d, f, h) under ruleset 1.0 for the four conflict scenarios. For Mali 2012 in Fig. 6a, both Flee (predicted conflict [RF-daily]) and Flee (predicted conflict [RF-onset]) overestimate the number of arrivals in camps after the initial days of the simulation, while Flee (recorded conflict) underestimates during the majority of the simulation period. The ARD of Flee (predicted conflict [RF-daily]) fluctuates more significantly compared to Flee (predicted conflict [RF-onset]), particularly between days 50 and 150, reaching 0.9, as shown in Fig. 6b. Flee (predicted conflict [RF-onset]) performs better than Flee (recorded conflict), with lower or similar ARD values for most of the simulation period.

In Burundi 2015 situation (Fig. 6c), Flee (predicted conflict [RF-onset]) and Flee (recorded conflict) significantly underestimate the number of arrivals in camps during the early and middle simulation period and overestimate the numbers at the later stage of simulation, while Flee (predicted conflict [RF-daily]) significantly underestimates the number of arrivals in camps during the whole simulation period. As can be seen in Fig. 6d, Flee (predicted conflict [RF-onset]) performs better than Flee (recorded conflict), with lower ADR during most of the days in the simulation, while Flee (predicted conflict [RF-daily]) performs worse than Flee (recorded conflict) during the middle and later simulation period (after around day 180).

For South Sudan 2013 (Fig. 6e), Flee (predicted conflict [RF-onset]) predicts a lower number of arrivals in camps than observational data from UNHCR at the early stage of simulation and overestimates the numbers afterwards, while Flee (predicted conflict [RF-daily]) predicts lower numbers for most of the simulation period. Flee (recorded conflict) predicts a much lower number of arrivals in camps than the UNHCR data at the beginning of the simulation, and then the difference between the predicted numbers and the UNHCR data becomes less. Fig. 6f shows that Flee (predicted conflict [RF-daily]) achieves lower ARD values than Flee (recorded conflict) during most of the simulation period, achieving ARD values below 0.5 after around day 250. Although Flee (predicted conflict [RF-onset]) obtains higher ARD values (0.8-1) in the initial stage, the ARD decreases significantly afterwards and maintains an ARD lower than 0.2 from the middle stage of the simulation. Flee (recorded conflict) performs the worst, with ARD values above 0.5 for the first half of the simulation.

In CAR 2013 situation, as shown in Fig. 6g and h, Flee (predicted conflict [RF-onset]) predicts similar results with Flee (recorded conflict), but with slightly higher ARD values. Flee (predicted conflict [RF-daily]) predicts a lower number of arrivals in camps than the UNHCR data during the whole simulation period, with higher ARD values than the other two models for most of the simulation period. Among the three simulation instances, Flee (recorded conflict) achieves the lowest ARD values for most of the simulation period. Flee (predicted conflict [RF-daily]) and Flee (predicted conflict [RF-onset]) achieve ARD values lower than 0.5 for about two-thirds of the simulation period, but Flee (predicted conflict [RF-onset]) achieves much lower ARD values than Flee (predicted conflict [RF-daily]). The main reason for the lower accuracy of the coupled model on this conflict instance could be that the prediction of conflicts becomes less accurate when predicting for a longer time.

## Conclusion

In this paper, we have developed a novel approach for predicting real-world conflict-driven displacement. By combining machine learning-based conflict forecasting with ABM, the coupled model automatically generates potential future conflict progression, eliminating the need for expert estimates. The RF model has been employed for designing conflict forecasting models, and two types of predicted conflict progress as output have been integrated into the Flee ABM to forecast the number of camp populations in neighboring countries. We have conducted experiments on four historical conflicts with different simulation periods, and the accuracy of simulations has been validated using the ARD metric. The ARD results have shown that using the Flee ABM to forecast without ACLED data is certainly feasible, and the coupled model has demonstrated comparable predictive accuracy versus the original Flee ABM on all tested conflict instances under two versions of simulation rulesets.

One might have assumed that the distortion in ARD in our migration forecasts would correlate somehow with the accuracy or confusion matrices of the conflict prediction validation. However, as far as we can tell, this is not clearly the case across these four conflicts, and a broader study would be required to investigate the possible existence of such a relation. If indeed there is no correlation between the accuracy of the conflict and the change in ARD of the migration forecasts, as our results seem to hint at, then there can be other causes that may lead to the discrepancy in ARD. For example, it is possible that ACLED did not accurately capture all major conflict events across the four conflicts, or that our encoding of the ACLED events into conflict input files is too simplistic. Another possible cause may be that the UNHCR data itself is not a complete reflection of the ground truth, as it is known to have omissions and can be subject to (political) bias. Given that the ARD actually decreases in 12 of our 16 simulation comparisons when adopting a (more approximate) conflict forecasting tool, we certainly need to remain aware of these possible causes.

Overall, this study emphasizes the potential of the proposed model in a real-world migration forecasting context, where ground truth conflict progression information does not exist and simulations cannot be done in a conventional way. There are several directions for future work. First, one limitation of the work we present here, and of the Flee ABM in general, is that we are not yet able to accurately estimate how *many* persons are displaced in a future conflict. However, being able to forecast the conflict itself constitutes an important step towards lifting this limitation, and indeed we intend to investigate this topic as part of our future work. Inclusion of the intensity of conflict is another potential source of improvement. Second, we plan to adopt additional evaluation metrics, such as the Mean Absolute Scaled Error (MASE)^10^, to further evaluate simulation accuracy across different conflict scenarios. Third, the current model assumes homogeneous (identical) agents. Future extensions of the model will incorporate demographic characteristics such as age, gender, and education level, along with different behavioral rules, to better capture the complexity and diversity of real-world displacement dynamics.

## Supplementary Information

Supplementary Information.

## Data Availability

The input and output data are publicly available on Figshare with DOI https://doi.org/10.17633/rd.brunel.28401116.v1, under a CC-By 4.0 license.

## References

[1] Lin, F. et al. The impact of russia-ukraine conflict on global food security. *Global Food Security* **36**, 100661 (2023).

[2] Rabbi, M. F., Ben Hassen, T., El Bilali, H., Raheem, D. & Raposo, A. Food security challenges in europe in the context of the prolonged russian-ukrainian conflict. *Sustainability* **15**, 4745 (2023).

[3] UNHCR. Figures at a glance 2024. United Nations High Commissioner for Refugees (2025). Available at: https://www.unhcr.org/about-unhcr/overview/figures-glance.

[4] Bonabeau, E. Agent-based modeling: Methods and techniques for simulating human systems. *Proceed. Nat. Academy Sci.* **99**, 7280–7287 (2002). 10.1073/pnas.082080899 PMC128598 12011407

[5] Macal, C. M. & North, M. J. Tutorial on agent-based modeling and simulation. In *Proceedings of the Winter Simulation Conference, 2005.*, 2–15 (IEEE, 2005).

[6] Abar, S., Theodoropoulos, G. K., Lemarinier, P. & O’Hare, G. M. Agent based modelling and simulation tools: A review of the state-of-art software. *Comput. Sci. Rev.* **24**, 13–33 (2017).

[7] Lemos, C., Coelho, H. & Lopes, R. J. Agent-based modeling of social conflict, civil violence and revolution: state-of-the-art-review and further prospects. In *Proceedings of the 11th European Workshop on Multi-Agent Systems (EUMAS 2013)* (CEUR-WS, 2013).

[8] Klabunde, A. & Willekens, F. Decision-making in agent-based models of migration: State of the art and challenges. *Eur. J. Population* **32**, 73–97 (2016). 10.1007/s10680-015-9362-0 PMC4803816 27069292

[9] Groen, D. Simulating refugee movements: Where would you go?. *Procedia Comput. Sci.* **80**, 2251–2255 (2016).

[10] Suleimenova, D., Bell, D. & Groen, D. A generalized simulation development approach for predicting refugee destinations. *Sci. Reports* **7**, 13377 (2017). 10.1038/s41598-017-13828-9 PMC5645318 29042598

[11] Suleimenova, D. & Groen, D. How policy decisions affect refugee journeys in South Sudan: A study using automated ensemble simulations. *Journal of Artificial Societies and Social Simulation* **23**, 10.18564/jasss.4193 (2020).

[12] Chadefaux, T. Conflict forecasting and its limits. *Data Sci.* **1**, 7–17 (2017).

[13] Von Soest, C. Why do we speak to experts? reviving the strength of the expert interview method. *Perspect. Politics* **21**, 277–287 (2023).

[14] Cederman, L.-E. & Weidmann, N. B. Predicting armed conflict: Time to adjust our expectations?. *Science* **355**, 474–476 (2017). 28154047 10.1126/science.aal4483

[15] Chadefaux, T. An automated pattern recognition system for conflict. *J. Comput. Sci.* **72**, 102074 (2023).

[16] Chadefaux, T. A shape-based approach to conflict forecasting. *Int. Interact.* **48**, 633–648 (2022).

[17] Muchlinski, D., Siroky, D., He, J. & Kocher, M. Comparing random forest with logistic regression for predicting class-imbalanced civil war onset data. *Polit. Anal.* **24**, 87–103 (2016).

[18] Schrodt, P. A. Seven deadly sins of contemporary quantitative political analysis. *J. Peace Res.* **51**, 287–300 (2014).

[19] Cederman, L.-E. & Vogt, M. Dynamics and logics of civil war. *J. Conflict Resol.* **61**, 1992–2016 (2017).

[20] No lessons learned from the holocaust? assessing risks of genocide and political mass murder since 1955. *American Political Science Review* **97**, 57–73. 10.1017/S0003055403000522 (2003).

[21] Goldstone, J. A. et al. A global model for forecasting political instability. *Am. J. Polit. Sci.* **54**, 190–208 (2010).

[22] Weidmann, N. B. & Ward, M. D. Predicting conflict in space and time. *J. Conflict Resol.* **54**, 883–901 (2010).

[23] Hegre, H. et al. Views: A political violence early-warning system. *J. Peace Res.* **56**, 155–174 (2019).

[24] Chadefaux, T. Early warning signals for war in the news. *J. Peace Res.* **51**, 5–18 (2014).

[25] Blattman, C. & Miguel, E. *Civil war. J. Econ. Literat.* **48**, 3–57 (2010).

[26] Sundberg, R. & Melander, E. Introducing the ucdp georeferenced event dataset. *J. Peace Res.* **50**, 523–532 (2013).

[27] Raleigh, C., Linke, R., Hegre, H. & Karlsen, J. Introducing ACLED: An armed conflict location and event dataset. *J. Peace Res.* **47**, 651–660 (2010).

[28] Mehrab, Z. *et al.* An agent-based framework to study forced migration: A case study of ukraine. *PNAS Nexus* **3**, pgae080, 10.1093/pnasnexus/pgae080 (2024). 10.1093/pnasnexus/pgae080 PMC10949908 38505694

[29] Zhang, W., Valencia, A. & Chang, N.-B. Synergistic integration between machine learning and agent-based modeling: A multidisciplinary review. *IEEE Trans Neural Networks and Learn. Syst.* **34**, 2170–2190 (2021). 10.1109/TNNLS.2021.3106777 34473633

[30] Dehkordi, M. A. E. *et al.* Using machine learning for agent specifications in agent-based models and simulations: A critical review and guidelines. *Journal of Artificial Societies and Social Simulation* **26** (2023).

[31] Monti, C., Pangallo, M., De Francisci Morales, G. & Bonchi, F. On learning agent-based models from data. *Sci. Reports* **13**, 9268 (2023). 10.1038/s41598-023-35536-3 PMC10247821 37286576

[32] Shuchami, A. & Lazebnik, T. Spatio-temporal sir model of pandemic spread during warfare with optimal dual-use healthcare system administration using deep reinforcement learning. arXiv preprint arXiv:2412.14039 (2024). 10.1017/dmp.2025.10062 40686043

[33] Zhang, Y., Grignard, A., Lyons, K., Aubuchon, A. & Larson, K. Real-time machine learning prediction of an agent-based model for urban decision-making. In *Proceedings of the 17th International Conference on Autonomous Agents and MultiAgent Systems*, 2171–2173 (2018).

[34] Schutte, S. & Weidmann, N. B. Diffusion patterns of violence in civil wars. *Polit. Geography* **30**, 143–152 (2011).

[35] Yurevich, P. A., Olegovich, M. A., Mikhailovich, S. V. & Vasilievich, P. Y. Modeling conflict in a social system using diffusion equations. *Simulation* **94**, 1053–1061 (2018).

[36] Ge, Q. et al. Modelling armed conflict risk under climate change with machine learning and time-series data. *Nat. Commun.* **13**, 2839 (2022). 35595793 10.1038/s41467-022-30356-x PMC9123163

[37] Bazzi, S. et al. The promise and pitfalls of conflict prediction: Evidence from Colombia and Indonesia. *The Rev. Econ. Stat.* **104**, 764–779. 10.1162/rest_a_01016 (2022).

[38] Schincariol, T. & Chadefaux, T. Temporal patterns in migration flows evidence from south sudan. *J. Forecast.* **44**, 575–588 (2025).

[39] Turkoglu, O. & Chadefaux, T. Nowhere to go? why do some civil wars generate more refugees than others?. *Int. Interact.* **45**, 401–420 (2019).

[40] Suleimenova, D., Low, W. & Groen, D. An agent-Based forced displacement simulation: A case study of the Tigray crisis. In *International Conference on Computational Science*, 83–89 (Springer, 2022).

[41] Groen, D., Suleimenova, D., Jahani, A. & Xue, Y. Facilitating simulation development for global challenge response and anticipation in a timely way. *J. Comput. Sci.* **72**, 102107. 10.1016/j.jocs.2023.102107 (2023).

[42] Groen, D. *et al.* Towards modelling the effect of evolving violence on forced migration. In *2019 Winter Simulation Conference (WSC)*, 297–307 (IEEE, 2019).

[43] Searle, C. & van Vuuren, J. Modelling forced migration: A framework for conflict-induced forced migration modelling according to an agent-based approach. *Comput., Environ. Urb. Syst.* **85**, 101568. 10.1016/j.compenvurbsys.2020.101568 (2021).

[44] Ghorbani, M. et al. Flee 3: Flexible agent-based simulation for forced migration. *J. Comput. Sci.* **81**, 102371 (2024).

[45] Anastasiadis, P. *et al.* P-flee: An efficient parallel algorithm for simulating human migration. In *2021 IEEE International Parallel and Distributed Processing Symposium Workshops (IPDPSW)*, 1008–1011 (IEEE, 2021).

[46] Wang, Y. Comparing random forest with logistic regression for predicting class-imbalanced civil war onset data: A comment. *Polit. Anal.* **27**, 107–110 (2019).

[47] Suleimenova, D., Arabnejad, H., Edeling, W. N. & Groen, D. Sensitivity-driven simulation development: a case study in forced migration. *Philos. Trans. Royal Soc. A* **379**, 20200077 (2021). 10.1098/rsta.2020.0077 PMC8059562 33775152

[48] Groen, D. et al. Fabsim3: An automation toolkit for verified simulations using high performance computing. *Comput. Phys. Commun.* **283**, 108596 (2023).

[49] Bosak, B. *et al.* Verification, validation and uncertainty quantification of large-scale applications with QCG-PilotJob. In *International Conference on Computational Science*, 495–501 (Springer, 2021).

