# Supplementary material for: Using machine learning to forecast conflict events for use in forced migration models
Source: Sci Rep. 2025 Aug 2;15:28202. doi: 10.1038/s41598-025-11812-2 (PMC12317072; doi:10.1038/s41598-025-11812-2)
Supplement: Supplementary file 1 — Supplementary Information. [file 41598_2025_11812_MOESM1_ESM.pdf]

The Supplementary Information of the Paper “Using machine learning to forecast conflict events for use in forced migration models”

Yani Xue, Thomas Schincariol, Thomas Chadefaux and Derek Groen

I. Simulation settings

For three simulation instances, we configure the parameter settings in `simsetting.yml`. In ruleset 1.0, we consider the following settings:

```
log_levels:
  agent: 0
  camp: 0
  init: 0
spawn_rules:
  conflict_driven_spawning:
    spawn_mode: "pop_ratio"
    displaced_per_conflict_day: 0.001
  take_from_population: True
  conflict_spawn_decay: [1.0,1.0,1.0,0.5,0.1]
move_rules:
optimisations:
```

Compared to ruleset 1.0, in ruleset 2.0, we modify `simsetting.yml` by adding several move rules:

```
log_levels:
  agent: 0
  camp: 0
  init: 0
spawn_rules:
  conflict_driven_spawning:
    spawn_mode: "pop_ratio"
    displaced_per_conflict_day: 0.001
  take_from_population: True
  conflict_spawn_decay: [1.0,1.0,1.0,0.5,0.1]
move_rules:
  start_on_foot: True
  avoid_short_stints: True
  use_pop_for_loc_weight: False
  awareness_level: 1
  weight_power: 0.8
optimisations:
```

Supplementary Table 1: Settings of simulation parameters by category

**log_levels**	**Type**	**Value**	**Justification**
agent	int	0	No information related to all agents movement is obtained from simulation runs
camp	int	0	No information related to camp is obtained from simulation runs
init	int	0	No information related to initialisation is obtained from simulation runs
**spawn_rules**	**Type**	**Value**	**Justification**
spawn_mode	string	"pop_ratio"	Spawn a certain percentage of the population in conflict zones
displaced_per_conflict_day	double	0.001	Spawned agents is 0.1% population in conflict zone during each time step (per day) of the simulation. Thus, the displaced population from all conflict zones increases proportionally per day.
take_from_population	boolean	True	Subtract spawned agents from populations in conflict zones and avoid the chance that a conflict spawn more people than they are in the conflict zone.
conflict_spawn_decay	array	[1.0,1.0,1.0,0.5,0.1]	An multiplier array describing the number of displaced persons gradual decline over three months after a conflict occurred.
**move_rules**	**Type**	**Value**	**Justification**
start_on_foot	boolean	True	Allow agents to traverse first link on foot
avoid_short_stints	boolean	True	Restrict displaced people who will take a break unless they have at least travelled for a full day's distance in the last two days.
use_pop_for_loc_weight	boolean	False	Exclude location population as a weighting factor for non-camp locations.
awareness_level	int	1	One hop in the location graph that an agent considers when planning a route and detecting suitable destinations.
weight_power	float	0.8	Agents will be more aggressive in dismissing suboptimal routes.

II. Conflict forecasting classification results

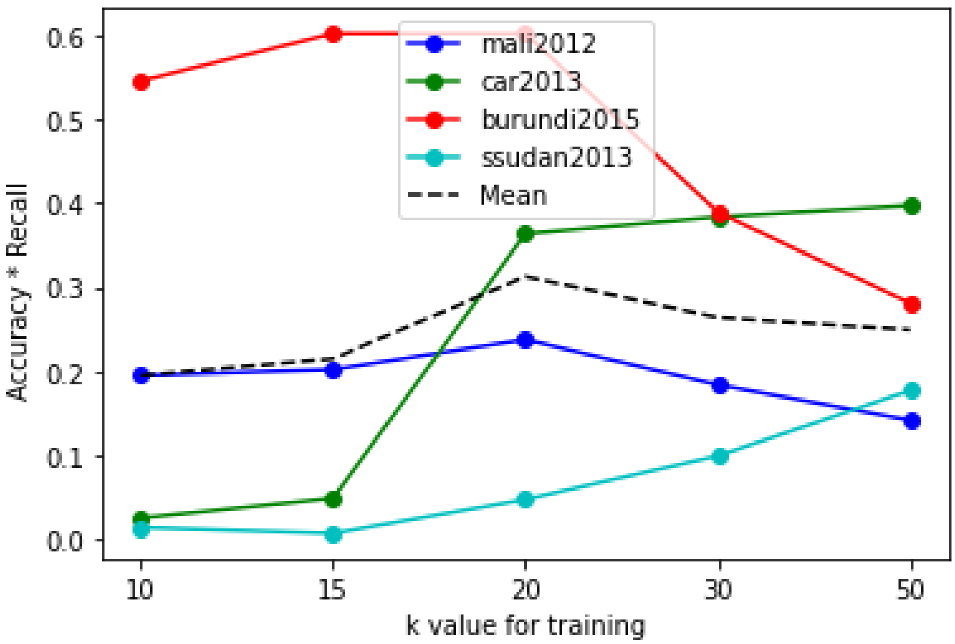

Supplementary Figure 1: The product of accuracy and recall for different downsampling factor.

Supplementary Table 2: Performance metrics for models across test cases.

Model	Case	Recall	Accuracy	ROC-AUC
RF	Mali-2012	0.293	0.812	0.475
	CAR-2013	0.425	0.855	0.653
	Burundi-2015	0.809	0.744	0.781
	SSudan-2013	0.051	0.936	0.494
	**Mean**	**0.394**	**0.837**	**0.601**
Random	Mali-2012	0.634	0.501	0.500
	CAR-2013	0.497	0.505	0.500
	Burundi-2015	0.542	0.499	0.500
	SSudan-2013	0.458	0.500	0.500
	**Mean**	**0.533**	**0.501**	**0.500**
Bernoulli	Mali-2012	0.024	0.980	0.500
	CAR-2013	0.017	0.971	0.500
	Burundi-2015	0.046	0.911	0.500
	SSudan-2013	0.022	0.948	0.500
	**Mean**	**0.027**	**0.952**	**0.500**

Supplementary Figure 2 shows the evolution of the F1-score through time for the four cases included in the study. As expected, the score decreases with time in the Burundi and CAR examples. In Mali and South Sudan, time brings more and more fluctuations in the F1-score, showing increasing uncertainty in the predictions. However, here, the objective is not primarily the conflict forecasting metrics, but rather to see if a conflict forecasting model is suited to produce inputs for the Flee model. Actually, the Flee model could be implemented for several periods, so the conflict model has to show comparable performances through different geographical conditions and timeframes.

Supplementary Table 3: Performance metrics for two models, RF and XGBoost, across test cases.

Model	Case	Recall	Accuracy	ROC-AUC
RF	Mali-2012	0.293	0.812	0.475
	CAR-2013	0.425	0.855	0.653
	Burundi-2015	0.809	0.744	0.781
	SSudan-2013	0.051	0.936	0.494
	**Mean**	**0.394**	**0.837**	**0.601**
XGBoost	Mali-2012	0.171	0.879	0.428
	CAR-2013	0.057	0.941	0.667
	Burundi-2015	0.534	0.601	0.597
	SSudan-2013	0.218	0.826	0.532
	**Mean**	**0.245**	**0.812**	**0.556**

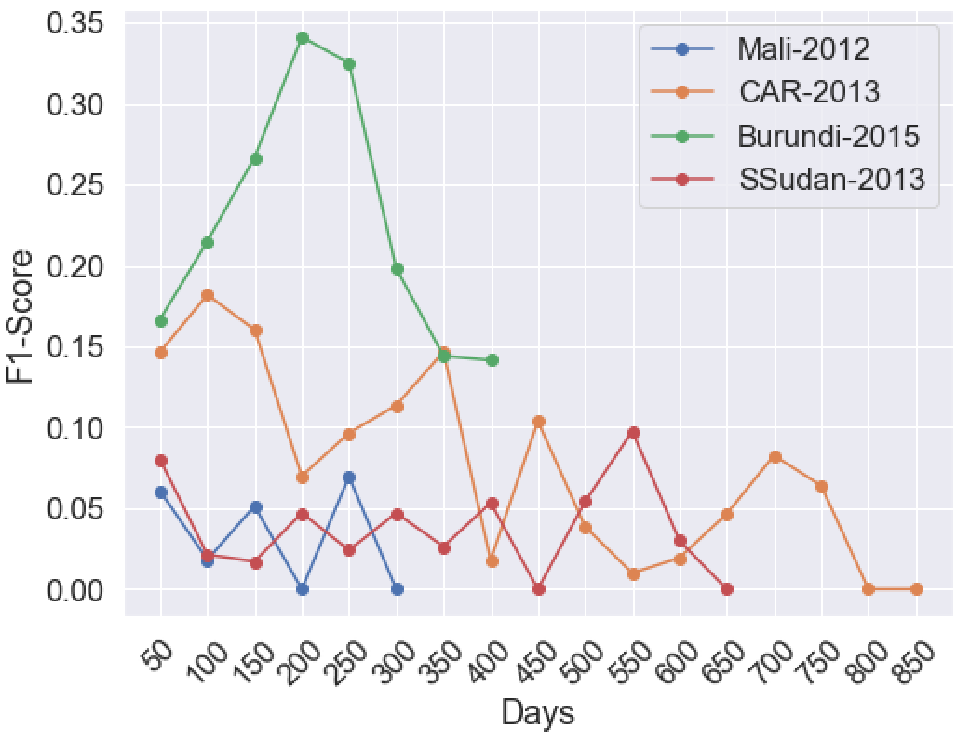

Supplementary Figure 2: F1-score (y-axis) through time (x-axis) for the four cases included in the study of the conflict forecasting model.

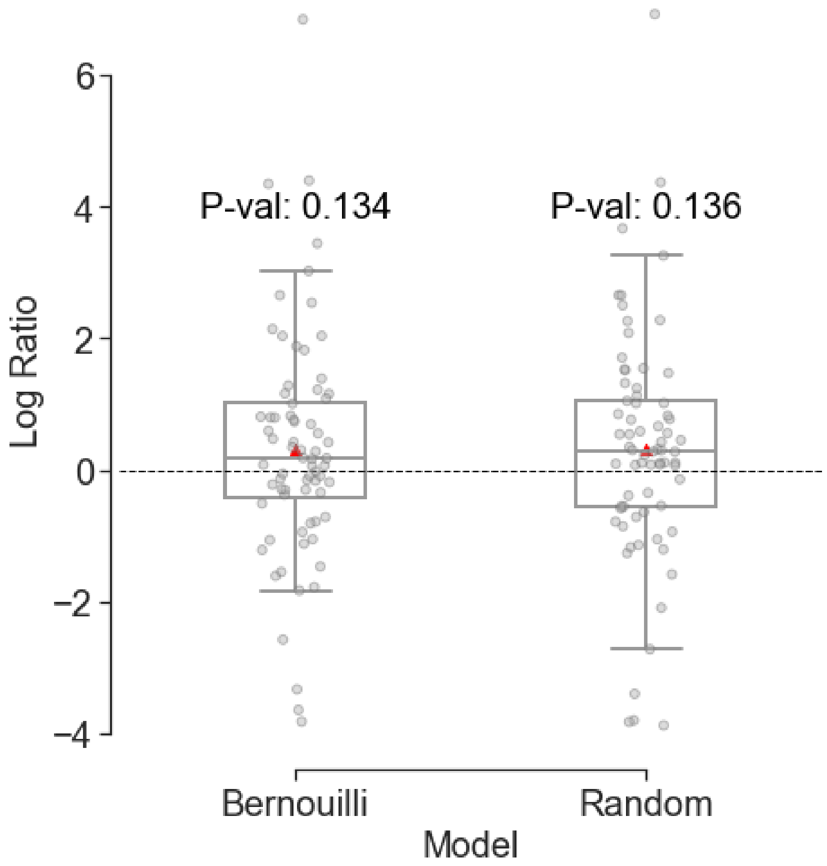

Supplementary Figure 3: Boxplot comparing the log ratios of two models, Bernoulli and random guess with the XGBoost model. Each point represents an individual data sample. The red triangles denote the mean value of MSE log ratio for each model. The p-values indicate the statistical significance of the difference between the log ratios for each model from 0. A positive log ratio implies a higher MSE, and worse performance for the benchmark model compared to our model.
